# Ribonuclease A Homologues of the Zebrafish: Polymorphism, Crystal Structures of Two Representatives and their Evolutionary Implications

**DOI:** 10.1016/j.jmb.2008.04.070

**Published:** 2008-06-27

**Authors:** Konstantina Kazakou, Daniel E. Holloway, Stephen H. Prior, Vasanta Subramanian, K. Ravi Acharya

**Affiliations:** Department of Biology and Biochemistry, University of Bath, Claverton Down, Bath BA2 7AY, UK

**Keywords:** 2′,5′-ADP, adenosine-2′,5′-diphosphate, Ang, angiogenin, Amph-2, Amphinase-2 from *Rana pipiens*, d(CpA), 2′-deoxy(cytidylyl-3′,5′-adenosine), 6-FAM-dArUdAdA-TAMRA, 6-carboxyfluorescein-dArUdAdA-6-carboxytetramethylrhodamine, hAng, human angiogenin, hECP, human eosinophil cationic protein, hEDN, human eosinophil-derived neurotoxin, LHM, loop-based Hausdorff measure, mAng-4, murine angiogenin-4, ONC, Onconase from *Rana pipiens*, Pca, pyrrolidone carboxylic acid (pyroglutamate), PDB, Protein Data Bank, PEG, poly(ethylene glycol), RC-RNase, *Rana catesbeiana* ribonuclease, RNase A, bovine pancreatic ribonuclease A, ribonuclease, angiogenin, X-ray crystallography, protein evolution, zebrafish

## Abstract

The widespread and functionally varied members of the ribonuclease A (RNase A) superfamily provide an excellent opportunity to study evolutionary forces at work on a conserved protein scaffold. Representatives from the zebrafish are of particular interest as the evolutionary distance from non-ichthyic homologues is large. We conducted an exhaustive survey of available zebrafish DNA sequences and found significant polymorphism among its four known homologues. In an extension of previous nomenclature, the variants have been named RNases ZF-1a–c,-2a–d,-3a–e and-4. We present the first X-ray crystal structures of zebrafish ribonucleases, RNases ZF-1a and-3e at 1.35-and 1.85 Å resolution, respectively. Structure-based clustering with ten other ribonuclease structures indicates greatest similarity to mammalian angiogenins and amphibian ribonucleases, and supports the view that all present-day ribonucleases evolved from a progenitor with three disulphide bonds. In their details, the two structures are intriguing melting-pots of features present in ribonucleases from other vertebrate classes. Whereas in RNase ZF-1a the active site is obstructed by the C-terminal segment (as observed in angiogenin), in RNase ZF-3e the same region is open (as observed in more catalytically efficient homologues). The progenitor of present-day ribonucleases is more likely to have had an obstructive C terminus, and the relatively high similarity (late divergence) of RNases ZF-1 and-3 infers that the active site unblocking event has happened independently in different vertebrate lineages.

## Introduction

Pyrimidine-specific ribonucleases with homology to bovine pancreatic ribonuclease A (RNase A; EC 3.1.27.5) are remarkably widespread among vertebrates. Representatives have been isolated from mammals, birds, amphibians, reptiles and, most recently, fish.^1–4^ Many of these enzymes are endowed with special biological activity(ies); for example, some stimulate the development of vascular endothelial cells,^2,5,6^ dendritic cells,^7^ and neurons,^8,9^ are cytotoxic/anti-tumoral^10^ and/or anti-pathogenic.^11^ In some cases, this is dependent on catalytic activity, but in others, it is not. In view of these features, the RNase A superfamily provides an excellent opportunity to study evolutionary forces at work on a conserved protein scaffold.

Current structural data indicate that catalytically active members of the superfamily typically comprise a polypeptide chain of 119 ± 15 residues, have an α + β topology with three conserved disulphide bond positions (SCOP^12^ code 54076), and employ a catalytic triad of residues (two His, one Lys) to cleave RNA on the 3′-side of pyrimidine nucleotides.^13^ Structural diversity comes in several forms. Some of these are more biochemical in nature, such as the oligomeric state^14^ or surface charge density,^11^ while others are more subtle, such as where the modification of loop regions alters the fine structure of the active site. Here, evolutionary forces have refined the ribonucleolytic performance of different members. Importantly, all the aforementioned forms of structural variation are known to influence biological activity.

Recent surveys of the data from zebrafish cDNA and genome sequencing projects have led to the identification and characterisation of RNase A homologues in this species.^2,3^ The enzymes have varying ribonucleolytic strength and are endowed variously with angiogenic and anti-bacterial activities. This, allied with sequence homology to mammalian angiogenin (Ang), has led to their classification as Ang-like and has been used to support the view that all present-day RNases evolved from Ang-like progenitors.^2,3,15^ In the present study, we reinvestigate the available sequence data and find that, in line with the high degree of genetic variation in this species,^16^ zebrafish ribonucleases exhibit significant polymorphism. At an early point in this investigation (and before the publication of the first report on zebrafish ribonuclease sequences),^2^ we selected a range of variants for further study. We present here the X-ray crystal structures of two representatives that yielded high-resolution data. Through systematic comparison of the most divergent regions of structure, we generate further evidence for the evolutionary origins of present-day ribonucleases. Most importantly, the structures reveal the three-dimensional details of relationships with other enzymes that are not prominent in amino acid sequence alignments and illustrate the assignment of kinship.

## Results

### Polymorphism

**Fig. 1:**
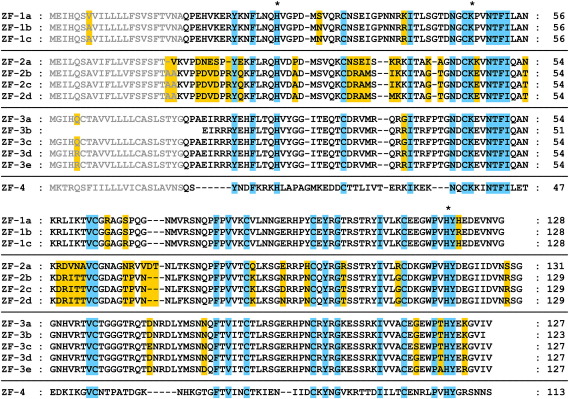
Alignment of the sequences of zebrafish RNase variants. Sequences were deduced from the sources given in Table 1 (nucleic acids encoding RNases ZF-1a–c,-2b–2d,-3d and-3e were resequenced during the course of this work). Residues predicted to form the signal peptide and mature chain of each protein are written in grey and black text, respectively. Residues conserved throughout are shaded blue, while those that vary within each subclass are shaded gold. Likely members of the catalytic triad are denoted by asterisks.

The combined efforts of two research groups have identified four nucleotide sequences encoding homologues of RNase A in the zebrafish (RNases ZF-1–4).^2,3^ The tissues and breeding lines from which these sequences were derived are varied, which prompted us to conduct an exhaustive survey of available zebrafish sequences. For RNases ZF-1,-2 and-3, we identified three, four and five variants, respectively, which we designate 1a–c, 2a–d and 3a–e (an extension of the nomenclature used by Pizzo and D’Alessio^17^), while for RNase ZF-4, we could not add to the single known form (Table 1; Fig. 1). The set of sequences includes representatives from the Tübingen (TU), AB, SJD and Singapore wild-type lines,^18^ and gives an indication of the variation within and between the different lines. Throughout, signal peptide cleavage sites predicted with SignalP 3.0 were consistent with those reported by Cho and Zhang,^3^ but were three or four residues upstream of those suggested by Pizzo *et al.*^2^

A gene encoding RNase ZF-1 is yet to be found in the genome assembly (version 7 at the time of writing), and corresponding sequences are currently restricted to cDNA libraries. RNase ZF-1a is the clone identified by Pizzo *et al.*^2^ in RZPD 609 (also known as MPMGp609), a library constructed from multiple tissues extracted from multiple, non-inbred individuals.^19^ RNases ZF-1b and-1c are encoded by clones present in a library prepared from an SJD embryo. We obtained I.M.A.G.E. Consortium clones of RNases ZF-1a–c and found that the deposited sequences were incomplete, lacking five or six residues at the C terminus. RNases ZF-1b and-1c are more similar to each other (two mismatches) than to RNase ZF-1a (four mismatches).

The sequence of RNase ZF-2a corresponds to that reported previously,^2,3^ is present in the genome assembly and is well represented in the non-normalised cDNA libraries FDR107 and NIH_ZGC_8. RNase ZF-2b is found in a single instance in two independent libraries, while RNases ZF-2c and-2d are single-copy members of NIH_ZGC_8. RNase ZF-2a differs considerably from RNases 2b–d (30 mismatches) which are closely related to one another (two variable positions).

RNase ZF-3a is encoded by a gene in the genome assembly, while RNases ZF-3b and-3c correspond to the cDNA sequence variants obtained by Pizzo *et al.*^2^ and Cho and Zhang (“*Dr*-RNase 1”)^3^ using an unspecified breeding line and the AB line, respectively. RNase ZF-3d is well represented in cDNA libraries prepared from the TU and AB lines, and is present in the Singapore line, while RNase ZF-3e was detected only in the Singapore line. Among this group, the mature protein sequence shows six positions of variance.

The sequence of RNase ZF-4 was deduced by Cho and Zhang from DNA sequences obtained from the TU and AB lines (“*Dr*-RNase 3”).^3^ We found no corresponding cDNA or additional sequence information in the databases.

### Ribonucleolytic activity

**Fig. 2:**
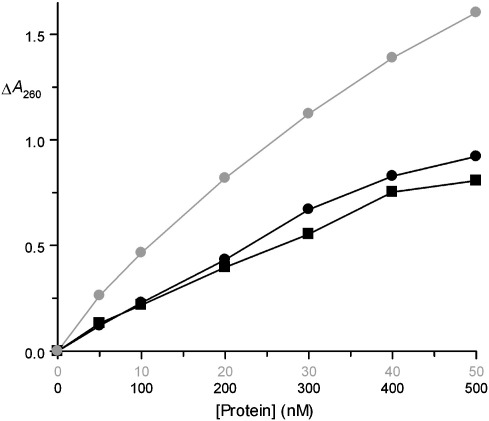
Comparative tRNA cleavage activities. Assays measured the release of perchloric acid-soluble fragments catalysed by RNase ZF-1a (●), RNase ZF-3e () and hAng (▪) as described in Materials and Methods. Each datum point represents the mean of four or five measurements. In all cases, the standard deviation is less than 5% of the mean.

RNase ZF-1a cleaves tRNA with a specific activity that is almost identical with that of human angiogenin (hAng) (Fig. 2), i.e. some four to five orders of magnitude lower than that of RNase A. This is the first report of its activity toward a polymeric substrate. The relative activities of RNase ZF-1a and hAng are slightly different from those measured for the cleavage of 6-carboxyfluorescein-dArUdAdA-6-carboxytetramethylrhodamine (6-FAM-dArUdAdA-TAMRA; a short fluorogenic substrate), where the specific activity of RNase ZF-1a is 3-fold greater.^2^ RNase ZF-3e is 17–20-fold more active than RNase ZF-1a or hAng toward tRNA. This is approximately double the figure reported previously for the cleavage of yeast tRNA by RNase ZF-3c,^3^ or the cleavage of 6-FAM-dArUdAdA-TAMRA by RNase ZF-3b.^2^

In the natural host, RNases ZF-1a and-3e most likely possess an N-terminal pyroglutamate residue (Pca1). The proteins assayed here differ in that Pca1 is replaced with Met(–1)–Gln1. When such a replacement is made in hAng, there is no change in ribonucleolytic activity^20^ owing to the presence of a relatively long N-terminal segment that distances Pca1 from the active site; the same is expected for RNases ZF-1a and-3e. Experiments described below employed forms of RNases ZF-1a and-3e possesing an N-terminal His-tag. Their specific activities differed from those of their Met(–1) counterparts by no more than 30% (data not shown). Since the formation of the catalytic site in an RNase A homologue requires the precise alignment of several non-contiguous structural elements, this indicates that the tags do not have a major impact on the overall protein conformation.

### Quality of the crystal structures

His-tagged forms of RNases ZF-1a and-3e were crystallised in the presence of high-molecular mass poly(ethylene glycol) (PEG) at pH 8–8.5, yielding monomeric structures at resolutions of 1.35 Å and 1.85 Å, respectively. The final models comprise residues (–1)–126 and 5–127, respectively (negative residue numbers denote those belonging to the His tag). Both His tags are significantly disordered, as are the C-terminal residue of RNase ZF-1a and the N-terminal four residues of RNase ZF-3e, but elsewhere the main chain is well ordered. Ramachandran plots indicate that the proportions of non-Pro/Gly residues falling in the most favoured regions are 91.6 % and 90.7 %, respectively, with the remainder in the other allowed regions.

### Topological overview and structure-based clustering

**Fig. 3:**
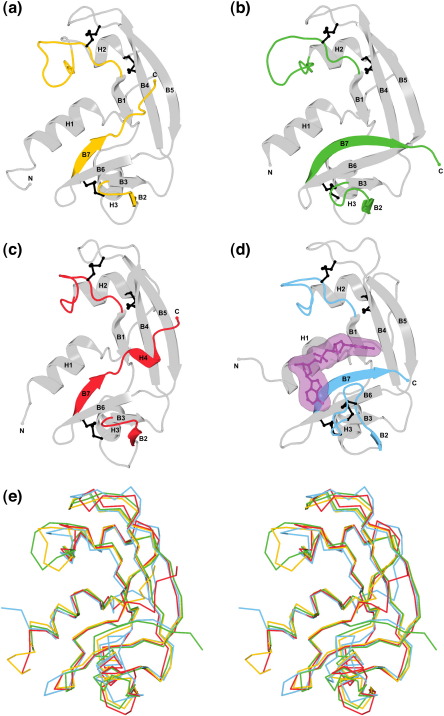
Topological comparison. Ribbon diagrams of (a) RNase ZF-1a, (b) RNase ZF-3e, (c) hAng (PDB entry 1B1I)^21^ and (d) RNase A·d(CpA) (PDB entry 1RPG)^22^, the latter including ball-and-stick and space-filling representations of the dinucleotide inhibitor. Elements of secondary structure are labelled, as are the N-and C-terminal extremities of each structure. Disulphide bonds are shown in black in ball-and-stick form. Several regions (strands B2 and B7, the segments immediately downstream, and the H2–B1 loop) are of particular use in comparing the four proteins and are highlighted in colour. (e) Stereo superposition of the C^α^ traces of the four proteins obtained with CE-MC.^82^ Colours correspond to the highlighted regions in (a–d).

The main chain of both structures adopts the characteristic bi-lobed α+β-fold of the pancreatic ribonuclease superfamily (Fig. 3a and b). The two C^α^ atom traces are more similar to each other (rms deviation = 1.1 Å for 115 equivalent atoms) than to any other structure in the Protein Data Bank (PDB). Within the PDB, the best global matches are made with orthologues of RNase 5 (e.g. hAng) and RNase 1 (e.g. RNase A), which are of mammalian origin (Fig. 3c–e). When aligned pairwise with hAng (PDB entry 1B1I),^21^ RNases ZF-1a and-3e deviate by 1.6 Å (rms) over 112 and 108 equivalent atoms, respectively; with RNase A (PDB entry 1RPG),^22^ the corresponding figures are 1.5 Å and 1.8 Å over 107 and 112 equivalent atoms.

**Fig. 4:**
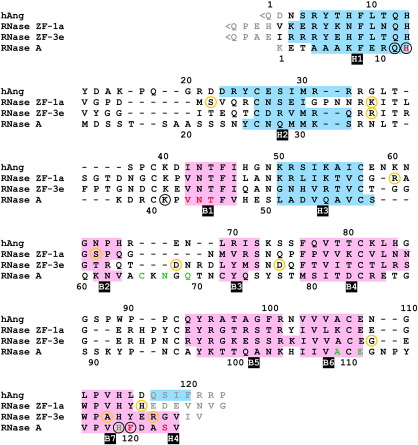
Structure-based sequence alignment. The C^α^ coordinates of RNase ZF-1a, RNase ZF-3e, hAng (PDB entry 1B1I)^21^ and RNase A·d(CpA) (PDB entry 1RPG)^22^ were aligned with CE-MC.^82^ Elements of secondary structure are shaded (α-and 3_10_-helices, blue; β-strands, pink) and labelled below. In the RNase A sequence, residues shown crystallographically to form the B_1_ and B_2_ subsites are coloured red and green, respectively, while those that form the P_1_ subsite are ringed in black. In the RNase ZF-1a and-3e sequences, positions that show polymorphism are ringed in gold. Numbering schemes for hAng and RNase A are given above and below the sequences, respectively. Residues that did not align or are not present in the respective crystal structures are written in grey text. < Q denotes a pyroglutamate residue.

**Fig. 5:**
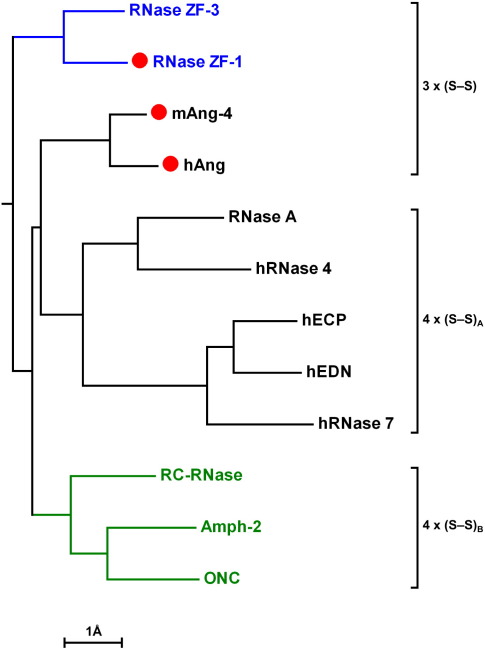
Structural relationships among ribonucleases. C^α^ coordinates of RNase ZF-1a, RNase ZF-3e, mAng-4 (PDB entry 2J4T, chain A),^30^ hAng (PDB entry 1B1I),^21^ RNase A·d(CpA) (PDB entry 1RPG),^22^ hRNase 4 (PDB entry 1RNF, chain A),^37^ hECP·2′,5′-ADP (PDB entry 1H1H),^24^ hEDN (PDB entry 1GQV),^23^ hRNase 7 (PDB entry 2HKY, model no. 15),^25^ RC-RNase·d(ACGA) (PDB entry 1M07, chain A),^27^ Amph-2 (PDB entry 2P7S)^28^ and ONC (PDB entry 1ONC)^26^ were aligned with CE-MC,^82^ and a loop-based Hausdorff measure (LHM) of structural dissimilarity^31^ was used to compute a pairwise distance matrix. A clustering tree was then constructed using the UNJ method,^83^ and rooted by reference to the fossil record.^85^ Branch lengths are scaled according to LHM distance; measured and reconstructed distances differ by 0.23 Å (mean) and peak at 0.66 Å (RNase ZF-3 *versus* RNase 7). Proteins with similar disulphide-bonding patterns are bracketed (the subscripted letters denote alternative 4 × (S–S) arrangements), while those that have obstructed B_1_ subsites and are angiogenic are marked with a red circle. Ichthyic, mammalian and amphibian clades are coloured blue, black and green, respectively.

Although the RNase A superfamily presents a highly conserved core topology, its constituent clades are defined to a large degree by the structures of extraneous regions such as loops and the fringes of the secondary structure elements. A cursory inspection of the available structures reveals that the zebrafish RNases have a characteristically long H2–B1 loop (Figs. 3 and 4); human eosinophil-derived neurotoxin (hEDN), human eosinophil cationic protein (hECP) and hRNase 7 have an unusually long B6–B7 loop;^23–25^ amphibian RNases have a general economy of loops;^26–28^ and the C-terminal segment of angiogenins is obstructive to substrate binding.^21,29,30^ With this in mind, we sought to identify the three-dimensional relationships among RNase ZF-1a, RNase ZF-3e and a representative set of ribonucleases through alignment of their C^α^ coordinates and application of a loop-based Hausdorff measure (LHM) of structural dissimilarity.^31^ The measure was applied to eight variable segments, most being loops but some having the potential for well-defined secondary structure, e.g. those corresponding to strand B2 and the C-terminal portion of strand B7 in RNase A. The LHM scores ranged from 1.45–8.01 Å and the resulting pairwise distance matrix enabled the construction of a clustering tree (Fig. 5). The tree indicates that RNases ZF-1 and-3 are more similar to amphibian RNases and angiogenins than to other mammalian ribonucleases such as RNase A, which is consistent with previous sequence-based phylogenetic analyses.^2–4^ The tree also supports a pathway for the emergence of the known disulphide-bonding patterns in which all present-day RNases derive from a progenitor with three disulphide bonds.^15^

The high resolution of the present structures permits a more detailed analysis of their structural and evolutionary links to other ribonucleases. The region that offers the greatest resource of structure-function data is the active site.^13^ Its central portion is conventionally divided into three subsites designated P_1_, B_1_ and B_2_, which bind the scissile phosphodiester linkage of the RNA substrate and the nucleotide bases located immediately upstream (a pyrimidine) and downstream (preferentially a purine), respectively.^32^ For all known RNase A homologues, these subsites are formed by residues located on opposing sides of the cleft between the two lobes (Fig. 3d). Across the superfamily, one of the lobes (composed principally of the B2–B3–B6–B7 β-sheet and its associated loops) exhibits structural variations that impact significantly on enzymatic performance and contribute to novel biological functions. Of particular interest is the β-hairpin formed by strands B2 and B3 at one edge, and strand B7 and the short C-terminal segment at the other. These regions are described in detail below.

### P_1_ subsite and catalytic centre

The structure of the P_1_ subsite is strongly conserved between RNase A and the zebrafish enzymes. It follows that the catalytic triad of RNase ZF-1a comprises His16, Lys47 and His119, while other phosphate-binding interactions are likely to be made by the side-chain amide group of Gln15 and the main chain N atom of Tyr120 (Fig. 4). In RNase ZF-3e, the corresponding residues are His16, Lys45 and His120, plus Gln15 and Tyr121. Typically, the C-terminal catalytic histidine adopts two main conformations, designated *A* (productive) and *B* (non-productive).^33^ Only *A* is observed for His119 of RNase ZF-1a, while a 50/50 mixture of *A* and *B* is observed for His120 of RNase ZF-3e. The absence of conformation *B* in RNase ZF-1a appears to be due to the crystal packing arrangement, which forces the side chain of Arg8 into the space occupied by conformation *B*. In neither of the zebrafish RNases is there any intramolecular interaction that might stabilise conformation *A*. This contrasts with the most efficient catalysts of the pancreatic ribonuclease superfamily such as RNase A, in which a hydrogen bond between His119 and Asp121 makes a 10^2^-fold contribution to *k*_cat_/*K*_m_.^34^

### B_1_ subsite and C-terminal segment

Structural studies on mammalian and amphibian RNases in complex with cytidylic and uridylic inhibitors indicate that the B_1_ subsite is a well-conserved, close-fitting pocket that makes two or three specific interactions with the base.^22,27,35–38^ Each of the present structures displays an obvious homology with previously reported structures in which the bulk of the pocket lining is formed by a universally conserved set of residues: in RNase ZF-1a, His16, Val49, Asn50, Thr51 and Tyr120; in RNase ZF-3e, His16, Val47, Asn48, Thr49 and Tyr121 (the corresponding residues of RNase A are His12, Val43, Asn44, Thr45 and Phe120) (Fig. 4).

**Fig. 6:**
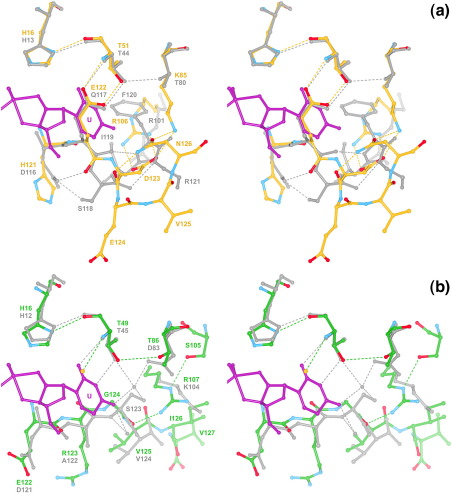
B_1_ subsite and C-terminal segment. RNase ZF-1a, RNase ZF-3e and hAng (PDB entry 1B1I)^21^ were aligned with the RNase A·uridine vanadate complex (PDB entry 1RUV)^86^ on the basis of the C^α^ positions of His12, Lys41, Thr45 and His119 in the latter. Shown in stereo are: (a) RNase ZF-1a (carbon, gold; nitrogen, blue; oxygen, red) superposed with hAng (grey); and (b) RNase ZF-3e (carbon, green; nitrogen, blue; oxygen, red) superposed with RNase A (grey). In both panels, the uridine vanadate moiety is shown in purple (pyrimidine ring labelled). A chloride ion in the RNase ZF-3e structure and two water molecules in the RNase A·uridine vanadate structure are shown as gold and grey spheres, respectively. Residue labels are coloured in accordance with the colouring of carbon atoms. Broken lines denote hydrogen bonds. The side chain of hAng Arg121 is omitted for clarity.

Thr51 of ZF-RNase-1a and Thr49 of ZF-RNase-3e are positioned in the same way as Thr45 of RNase A and are therefore expected to be the primary functional components of their respective B_1_ subsites, forming a pair of hydrogen bonds with either cytosine or uracil (Fig. 6a and b). In RNase ZF-1a, the anticipated binding position of the pyrimidine is taken by the side chain of Glu122, which makes two hydrogen bonds with Thr51 (Fig. 6a; Table 2). This is reminiscent of hAng, in which a similar obstruction is posed by the corresponding residue, Gln117.^21,29^ One residue upstream of the obstructive residue, the main chain begins a deviation from the neighbouring β-strand that marks the beginning of a novel C-terminal conformation (Figs. 3a and 6a). There is no interaction between the two residues that flank the obstructive residue (His121 and Asp123), contrasting with the two hydrogen bonds that link the corresponding residues of hAng (Asp116 and Ser118). Furthermore, the Glu122–Asp123 peptide bond is flipped relative to its hAng counterpart, and the C-terminal segment bulges out from the main body of the protein rather than inwards as is the case with hAng. Overall, the C-terminal conformation is less obstructive than that found in hAng; notably, Val125 points away from the B_1_ subsite, while its hAng counterpart, Phe120, encroaches on the pyrimidine-binding zone. The segment is stabilised chiefly by a network of electrostatic and polar interactions involving the side chains of two residues from the C-terminal segment (Asp123 and Asn126) and two residues from the main body of the protein (Lys85 and Arg106) (Fig. 6a; Table 3). In this respect, it bears some resemblance to murine angiogenin-4 (mAng-4; PDB entry 2J4T),^30^ which features an electrostatic interaction between the side chain of Arg99 (the counterpart of Arg106) and the free carboxylate group of the C terminus.

In RNase ZF-3e, the B_1_ subsite is unobstructed and the position expected for the 2-keto group of a bound pyrimidine is occupied by a chloride ion from the crystallisation medium (Fig. 6b). This has also been observed for RNase A when crystallised in the presence of a high concentration of chloride ions, e.g. PDB entry 1RNX.^39^ The C-terminal segment follows a path similar to that of RNase A, i.e. strand B7 maintains an extended antiparallel link to strand B6 (Figs. 3b and d). This is further stabilised by a hydrogen bond between Gly124 and the side chain of Arg107 (Fig. 6b; Table 3). Arg123, the counterpart of hAng Gln117, is too large a residue to occupy the pyrimidine-binding pocket and its side chain is instead oriented toward the solvent. As such, it is very similar to the Arg/Lys residues found at the corresponding positions in hEDN, hRNase 7 and Amphinase-2 (Amph-2) (PDB entries 1GQV, 2HKY and 2P7S, respectively^23,24,28^).

The cytidine/uridine specificity of the B_1_ subsite varies greatly among pancreatic ribonuclease superfamily members and is known to be modulated by two means. First, the hydrogen-bonding selectivity of the main Thr residue can be influenced by a neighbouring residue and second, additional interactions with the substrate may be made by other residues. In RNase ZF-1a, Lys85 lies adjacent to Thr51 and is the counterpart of the modulatory residues Thr80 and Asp83 that are found in hAng and RNase A, respectively (Fig. 6a and b). No other known RNase structure features a Lys residue at this position. The N^ζ^ group of Lys85 makes no interaction with the side-chain hydroxyl group of Thr51 and is instead engaged in interactions with Asp123 and Asn126 of the C-terminal segment (Fig. 6a; Table 3). However, it is likely that substrate binding triggers a significant restructuring of the C-terminal segment. During this process, the side chain of Lys85 could become involved in substrate binding. It may form a hydrogen bond with Thr51 or one directly with the 4-keto group of uracil. The guanidino group of Arg106 may also interact with the 4-keto group of uracil, thus emulating Arg101 of RNase 4, a residue that contributes to the strong uridine preference of that enzyme.^37,40^

In RNase ZF-3e, Thr86 corresponds well with Thr80 of hAng (Figs. 6a and b), a residue known to promote uridine selectivity by weakening the interaction between the main Thr residue (Thr44) and the N3 atom of cytosine.^41,42^ The distance between the O^γ1^ atoms of Thr49 and Thr86 measures 3.42 Å in the present structure, suggesting that a weak hydrogen bond is present and that Thr86 plays a role similar to that of Thr80 in hAng. Arg107 (the counterpart of Arg101 in RNase 4/Arg106 in RNase ZF-1a ) makes two hydrogen-bonding interactions that orient its guanidino group away from the B_1_ subsite, reducing the likelihood of a role in substrate binding.

In neither of the zebrafish RNases is there a direct counterpart of Ser123 of RNase A, a residue that forms two energetically significant water-mediated hydrogen bonds with the 4-keto group of uridine.^43^

### B_2_ subsite and B2–B3 β-hairpin

**Fig. 7:**
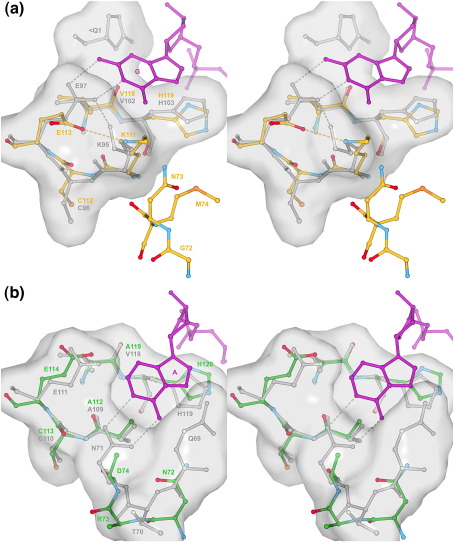
B_2_ subsite. RNase ZF-1a, RNase ZF-3e and the RC-RNase·d(ACGA) complex (PDB entry 1M07)^27^ were aligned with the RNase A·d(CpA) complex (PDB entry 1RPG)^22^ on the basis of the C^α^ positions of Ala109, Glu111 and His119 in the latter. Shown in stereo are: (a) RNase ZF-1a (carbon, gold; nitrogen, blue; oxygen, red) superposed with RC-RNase (grey); and (b) RNase ZF-3e (carbon, green; nitrogen, blue; oxygen, red) superposed with RNase A (grey). In each panel, a portion of dinucleotide (purple; purine ring labelled) and a surface representation of the subsite are contributed by the liganded structure. Residue labels are coloured in accordance with the colouring of carbon atoms. < Q1 denotes a pyroglutamate residue and broken lines denote hydrogen bonds. Conformation *B* of RNase ZF-1a Glu113, RNase ZF-3e His120 and RNase A Gln69 are omitted for clarity. RNase ZF-3e residues Arg73 and Asp74 are disordered beyond C^β^.

The ways in which adenylic and guanylic inhibitors are bound by various RNases^22,27,38,44,45^ infer that the B_2_ subsites of RNases ZF-1a and-3e have several familiar features in common. First, as is the case throughout the superfamily, the imidazole ring of the C-terminal catalytic histidine is positioned to make stacking interactions with the purine ring system (Fig. 7a and b). Second, Glu113 of RNase ZF-1a and Glu114 of RNase ZF-3e are poised to mimic Glu111 of RNase A/Glu97 of *Rana catesbeiana* ribonuclease (RC-RNase)/Glu91 of Onconase (ONC) and may, therefore, form hydrogen bonds with the N1 and/or N2 atoms of guanine. Third, any contribution from the loop of the B2–B3 β-hairpin is likely to be modest. In each case, the loop is fairly short (two residues in RNase ZF-1a, five in RNase ZF-3e) but includes at least one residue (Asn73 in RNase ZF-1a, Asn72 and Asp74 in RNase ZF-3e) that could conceivably interact with the heteroatom at position 6 of a bound purine, although this is most likely to be via a water molecule. Thus, this region is more elaborate than in amphibian RNases (where it is abridged) and is most akin to hAng where the loop comprises three residues (Fig. 3c). It contrasts with several mammalian enzymes such as RNases 1–4 and 7 where the loop is somewhat longer (eight residues in RNase A), is stabilised by an internal disulphide bond, and hydrogen-bonds directly to adenine (Figs. 3d and 7b).

There is one notable difference between the B_2_ subsites of the two zebrafish RNases. While RNase ZF-3e possesses an Ala residue at position 112 (a likely functional counterpart of Ala109, a simple pocket-lining residue of RNase A), RNase ZF-1a has a Lys residue at the corresponding position (residue number 111). The only precedent for this is Lys95 of RC-RNase. In the crystal structure of RC-RNase in complex with d(ACGA) (PDB entry 1M07), the side chain of Lys95 makes van der Waals interactions with guanine and stabilises the position of Glu97 *via* an N^ζ^–O^ε2^ hydrogen bond (Fig. 7a), thereby making a significant contribution to the guanine preference of the B_2_ subsite of that enzyme.^27^ Although in the present structure of RNase ZF-1a the side chain of Lys111 is obstructive to purine binding and appears to stabilise a wayward conformation of Glu113, substrate binding may trigger a rearrangement in which Lys111 and Glu113 come to mimic Lys95 and Glu97 of RC-RNase. Indeed, the two RC-RNase residues are also somewhat mobile, as indicated by their conformations in an unpublished nucleotide-free structure (PDB entry 1KM9).

## Discussion

Our survey of zebrafish sequences revealed significant polymorphism of RNases ZF-1–3. Although the inherent error rates of thermostable polymerases, reverse transcriptase and other procedures give rise to artifacts in all cDNA collections, there are several pointers as to the validity of the current analysis. First, the degree to which experimental artifacts contribute to the sequence variations observed in carefully constructed full-length cDNA libraries such as those of the Mammalian Gene Collection is low, at around 10%.^46^ Second, in several cases (e.g., RNases ZF-2a,-2b and-3b), representatives were found in multiple libraries, which would be highly unlikely to occur by chance. Third, we resequenced representative clones to ensure that the quality of the DNA sequencing was high. With regard to RNase ZF-1 (a copy of which is yet to be found in genomic DNA), each of the three variants that we identified must be taken as equally valid at the present time. In addition, the finding that RNases ZF-2b–d differ from the previously identified RNase ZF-2a at nearly one-quarter of all positions may necessitate the creation of a new RNase ZF-5 class.

Our structures of RNases ZF-1a and-3e are the first ichthyic ribonuclease structures to be reported. Classification using a loop structure-based dissimilarity measure produced a biologically meaningful tree (Fig. 5), contrasting with trees derived from measures of core similarity (not shown) and confirming the potential of this approach for obtaining systematic relationships from structural data.^47^ Regions that are not part of the conserved core tend not to be critical to the structural integrity of proteins and evolve mostly through insertion-deletion events.^48^ The tree suggests that this type of event has had a major role in the formation of the present-day ribonuclease clades and in the development of their special biological activities. Attributing statistical significance to particular branches of the tree is currently not possible. Character-based confidence tests, such as bootstrapping,^49^ are not appropriate, but analytical tests such as those based on weighted least-squares^50^ could be used if the error of the dissimilarity measure was known. However, this cannot be defined satisfactorily at present, as it depends on multiple factors including the resolution of the structures, the quality of the structural alignment and any disorder in the loop regions.

While sequence data suggest that all present-day ribonucleases descend from a progenitor with three disulphide bonds,^2–4,15^ the structure of RNase ZF-3e advances the debate. Its combination of three disulphide bonds and an unobstructive C terminus—which is also likely to be a feature of some reptilian and avian ribonucleases^6,51^—raises the question of whether the C terminus of the progenitor was obstructive or not. The clustering tree supports both possibilities (Fig. 5). In addition, the relatively high level of similarity (late divergence) of RNases ZF-1 and-3 infers that the unblocking/blocking event has happened independently in different vertebrate lineages. Since the low level of catalytic efficiency that results from an obstructive C-terminus is highly correlated with the presence of angiogenic activity, it seems most likely that the ancestral ribonuclease had an obstructive C terminus; the convergent evolution of angiogenic activity in vertebrate classes as distantly related as mammals and fish seems highly improbable in comparison.

Several regions of the new structures provide functional insight. In the region of the C-terminal segment, RNase ZF-1a bears an obvious (though incomplete) resemblance to hAng. The binding of RNA substrates to hAng requires a major structural rearrangement of this region,^52^ and the same appears to be true for RNase ZF-1a, which most likely underpins its low level of ribonucleolytic activity. The unobstructed B_1_ subsite of RNase ZF-3e identifies it not with hAng but with most of the other mammalian and all of the known amphibian ribonucleases. As expected, its ribonucleolytic activity is significantly greater than that of RNase ZF-1a or hAng. Since mutations that elevate ribonucleolytic activity are known to be detrimental to the angiogenic activity of mammalian angiogenins,^30,53,54^ this most likely accounts for the lack of angiogenic activity in its close relative, RNase ZF-3b.^2^ Its enzymatic prowess is still, however, several orders of magnitude lower than that of some relatives, e.g. RNase A. Contributions to this are likely to come from the lack of interactions stabilising the productive tautomer of His120 and the more rudimentary nature of its substrate-binding subsites.

At the B_1_ subsite of RNase ZF-1a, the position of Lys85 and the correspondence of Arg106 to Arg101 of RNase 4 suggest that this enzyme will bind uridine more avidly than cytosine,^37,40^ whereas in RNase ZF-3e, the positions of Thr86 and Arg107 suggest an ambivalence as for hAng.^41,42^ At the B_2_ subsite, predictions of base selectivity are hampered somewhat by the variable positioning and orientation of the purine when bound by different enzymes.^22,27,38,44,45^ The resemblance of RNase ZF-1a to RC-RNase suggests a preference for guanine over adenine here but the likely preference of RNase ZF-3e is unclear.

The 11 polymorphisms identified in RNases ZF-1 and-3 are spread over several structural elements (Fig. 4). The residues found at these positions are hydrophilic or small/non-polar and are all at exposed positions, eight being within loops. Most of the substitutions are unlikely to affect substrate binding or catalysis, although the three substitutions in the region of the second catalytic histidine and C-terminal segment may have small effects. It is possible that the Ala/Thr119 and Arg/Lys123 substitutions contribute to the apparent difference between the activities of RNase ZF-3e and-3b/c.

The wealth of ribonuclease crystal and solution structures permits some predictions about the structures of the remaining zebrafish enzymes. RNase ZF-2 is similar in length to RNases ZF-1 and-3 (Fig. 1). Its H2–B1 and B2–B3 loops are likely to be roughly the same length as those of RNase ZF-1a but its C-terminal segment is unusually long. If we define the position of the second catalytic histidine in the sequence as the origin, there is a Glu residue at +3 and large hydrophobic residues (both Ile) at +5 and +6. The same motif is found in hAng, suggesting that the C terminus of RNase ZF-2 obstructs the B_1_ subsite. This is consistent with the magnitude of its ribonucleolytic activity, which is small and similar to that of hAng.^2,3^ The specificity-determining elements of the B_1_ subsite appear similar to those of RNase ZF-3e, while those of the B_2_ subsite may be novel. In contrast, the sequence of RNase ZF-4 differs widely from those of RNases ZF-1–3. It is 14–16 residues shorter, with the majority of its deletions predicted to fall in loops such as those in the N-terminal, H2–B1 and B2–B3 segments. As in amphibian RNases such as ONC, the short N-terminal segment may permit the participation of Pca1 in catalysis,^55^ while the B2–B3 segment appears to be abridged. The C terminus is short and hydrophilic with an Arg residue at position +3. Therefore, the B_1_ subsite is likely to be unobstructed, as observed for RNase ZF-3e and other enzymes with an Arg/Lys in this position.^23,24,28^ This is consistent with its elevated ribonucleolytic activity.^3^ The specificity-determining elements of the B_1_ subsite appear to be novel but the presence of direct counterparts of ONC residues Thr89 and Glu91 suggest that the B_2_ subsite may be similar to that of the amphibian enzyme.^38^

## Materials and Methods

### Identification of zebrafish ribonuclease variants and selection of clones

Translated zebrafish nucleotide sequences held on the Zebrafish Information Network‡^56^ were first compared to known zebrafish ribonuclease amino acid sequences^2,3^ using TBLASTN.^57^ Follow-up searches of I.M.A.G.E. Consortium^58^ clustered expressed sequence tags were conducted using the IMAGEne server, v. 4.9.^59^ Sequence alignments were performed with ClustalW.^60^ Signal peptide cleavage positions were determined with SignalP 3.0.^61^

I.M.A.G.E. Consortium cDNA clones 3716043, 5628280 and 6907218, which encode RNases ZF-1a,-1b and-3e, respectively, were obtained from RZPD Deutsches Ressourcenzentrum für Genomforschung (Berlin, Germany). The plasmid inserts were sequenced from each end at MWG Biotech (London, UK) using appropriate vector-based primers. Clones 5627130, 7410112, 7264666, 7250488, 7047538 and 7151363, which encode RNases ZF-1c,-2b,-2c,-2d,-3d and-3d (again), respectively, were sequenced in the same manner at Geneservice Ltd (Cambridge, UK).

### Construction of expression strains

The coding sequences of mature RNases ZF-1a and-3e were amplified from the aforementioned cDNA clones by PCR with KOD Hot Start DNA polymerase (Novagen) in conjunction with the following primers: for RNase ZF-1a

5′-CATATGCAACCTGAACATGTAAAGGAGC-3′ (forward)
5′-CTCGAGTCAGCCTACGTTAACTTCGTCT-3′ (reverse) for RNase ZF-3e
5′-CATATGCAACCAGCAGAAATAAGGCG-3′ (forward)
5′-CTCGAGCTAAACAATAACACCTCTTTCATAGTG-3′ (reverse)

Amplification products were A-tailed with *Taq* polymerase/dATP and ligated with EcoRV-cleaved, T-tailed pGEM®-T Easy Vector (all Promega) to form pGEM-z1a and pGEM-z3e, respectively. The mixture was then used to transform *Escherichia coli* JM109 cells and authentic clones were identified by restriction digestion and DNA sequencing.

For crystallographic experiments, each protein was expressed with an N-terminal His_6_-containing tag. An RNase ZF-1a expression plasmid was created by use of a ligation-independent cloning PCR approach. The gene was first PCR-amplified from pGEM-z1a with the primers

5′-CACCACCACCACATGCAACCTGAACATGTAAAGGAGCG-3′ (forward)
5′-GAGGAGAAGGCGCGTTAGCCTACGTTAACTTCGTCTTCATGATAATGCAC-3′ (reverse)

The products were treated with phage T4 DNA polymerase (Novagen)/dATP to generate single-stranded overhangs and annealed with suitably prepared pET-YSBLIC^62^ (a pET-28a(+) derivative kindly donated by Dr M.J. Fogg, University of York, UK) to form pYSBLIC-z1a. For expression of RNase ZF-3e, the gene was excised from pGEM-z3e with NdeI and XhoI and ligated with similarly treated pET-15b (Novagen) to form pET15-z3e. The tagged proteins possessed N-terminal extensions of 11 and 21 residues, respectively.

For assay of ribonucleolytic activity, each protein was expressed in Met(–1) form, without a His tag. The sequence encoding RNase ZF-1a was PCR-amplified from pYSBLIC-z1a with the primers

5′-CATCACCACCACCATATGCAACCTGAACATGTAAAG-3′ (forward)
5′-GGAGAACTCGAGTTAGCCTACGTTAACTTCGTC-3′ (reverse)

and treated with NdeI and XhoI, while that encoding RNase ZF-3e was excised from pET15-z3e using the same enzymes. The DNA fragments were ligated with similarly digested pET-22b(+) (Novagen) to form pET22-z1a and pET22-z3e, and the absence of unintended mutation was established by DNA sequencing. These constructs do not make use of the *pelB* leader or His-tag encoded by pET-22b(+). All expression constructs were used to transform *E. coli* BL21-CodonPlus(DE3)-RIL cells (Stratagene).

### Purification of recombinant proteins

Recombinant proteins were prepared from expression strains as described.^63^ Briefly, strains were cultured at 37 °C in Terrific Broth^64^ supplemented with 0.5 % (w/v) glucose and appropriate antibiotic(s) (for pYSBLIC-z1a, 50 μg ml^− 1^ kanamycin and 25 μg ml^− 1^ chloramphenicol; for pET15-z3e, pET22-z1a and pET22-z3e, 100 μg ml^− 1^ ampicillin). Expression was induced by addition of isopropyl-β-D-thiogalactopyranoside to 1 mM, which directed the target proteins into inclusion bodies. These were solubilised and refolded, then purified by SP-Sepharose chromatography followed by C4 reversed-phase HPLC. Purified proteins were lyophilised and reconstituted in AnalaR grade water, at which point purity was > 98 % as judged by SDS-PAGE. Protein concentration was determined by measuring UV absorbance and using an estimated ε_280_^65^ of 13,325 M^− 1^ cm^− 1^ for all proteins.

Proteins were authenticated by electrospray ionisation time-of-flight mass spectrometry. Analyses were conducted by Dr A.T. Lubben (Department of Chemistry, University of Bath, UK) using a micrOTOF instrument (Bruker Daltonik). The recorded molecular masses of [Met(–1)]-RNase ZF-1a and-3e were 14679.3 Da and 14860.3 Da, respectively, which were within 1 Da of the predicted mass. Those recorded for His-tagged RNase ZF-1a (15,733.4 Da) and-3e (16,892.3 Da) were consistent with the presence of vector-encoded N-terminal extensions of sequence

GSSHHHHHHM and
MGSSHHHHHHSSGLVPRGSHM,

respectively, and were equally accurate.

### Ribonucleolytic activity assay

tRNA cleavage assays were conducted essentially as described,^66^ except that the buffer was 33 mM Na-Hepes (pH 7.0) and incubations were for 2 h. Assays contained 2 mg ml^− 1^ yeast tRNA (Sigma), 0.1 mg ml^− 1^ bovine serum albumin (Worthington Biochemical Corp.) and six concentrations of test protein. The reaction was terminated by the addition of 2 vol. ice-cold 3.4 % (w/v) perchloric acid and incubated on ice for 10 min. The mixture was centrifuged at 13,200 g for 10 min at 4 °C, after which the *A*_260_ of the supernatant was used as a measure of RNase activity. The magnitude of the activity in the most linear portion of each plot (three or four data points) was used in the calculation of relative activities. [Met(–1)]-hAng (available from a previous study^30^) and RNase A (Sigma) were used as reference samples and were found to have activities in accordance with previous measurements.^67^

### X-ray crystallography

Crystals of His-tagged RNase ZF-1a and-3e were grown at 16 °C by the hanging-drop, vapour-diffusion method. Protein solution (10 mg ml^− 1^ in water) was mixed with an equal volume of reservoir solution (for RNase ZF-1a, 25 % PEG 4000, 0.2 M (NH_4_)_2_SO_4_, 0.1 M Tris–HCl (pH 8.0); for RNase ZF-3e, 25 % PEG 4000, 0.2 M LiCl, 0.1 M Tris–HCl (pH 8.5)). RNase ZF-1a formed amorphous crystals within 3 days while RNase ZF-3e formed plate-like crystals within one week. Diffraction data from single crystals were collected on stations 14.2 (RNase ZF-1a, room temperature) and 10.1 (RNase ZF-3e, 100 K) of the Synchrotron Radiation Source (Daresbury, UK), each of which was equipped with a Quantum-4 CCD detector (Area Detector Systems Corp.). For RNase ZF-3e, the PEG 4000 concentration in the mother liquor was increased to 30 % to provide cryoprotection. All data were processed using the HKL suite^68^ and intensities were truncated to amplitudes using TRUNCATE.^69,70^ Matthews coefficients^71^ indicated that both types of crystal contained one molecule per asymmetric unit (for RNase ZF-1a, *V*_M_ = 1.9 Å^3^ Da^− 1^ and solvent content = 35 %, v/v); for RNase ZF-3e, *V*_M_ = 2.2 Å^3^ Da^− 1^ and solvent content = 45 %, v/v). Detailed data processing statistics are given in Table 3.

Similar approaches were used for determination of each structure. Initial phases were obtained using the molecular replacement routines of PHASER.^72^ For RNase ZF-1a, the search model was derived from the coordinates of [Met(–1)]-hAng (PDB entry 1ANG^29^) while for RNase ZF-3e, it was derived from those of RNase ZF-1a. The resultant models were refined using REFMAC5^73^ with 3 % (RNase ZF-1a) or 5 % (RNase ZF-3e) of reflections set aside for cross-validation.^74^ After an initial round of rigid-body refinement, rounds of restrained refinement were interspersed with electron density map calculations and manual adjustments using COOT.^75^ On the basis of *mF*_o_–*DF*_c_ electron density, side-chain atoms were omitted at some positions (RNase ZF-1a: Lys6; RNase ZF-3e: Ile5, Arg7, Arg8, Arg28, Arg73, Asp74) and dual conformations were modelled at others (RNase ZF-1a: Met(–1), Arg8, Gln15, Met21, Val23, Ser28, Thr38, Ser40, Asn56, Lys57, Ser69, Asn89, Glu98, Arg100, Thr102, Glu113; RNase ZF-3e: Val29, Val111, His120). Chloride ions were added manually, while water molecules were added using ARP-wARP^76,77^ at positions where *mF*_o_–*DF*_c_ electron density peaks exceeded 3σ and potential hydrogen bonds could be made. Model validation was conducted with the RCSB PDB Validation Suite^78^ and the WHAT_CHECK server.^79^ Detailed statistics for each model are given in Table 3. Figures were drawn with PyMOL§ (DeLano Scientific, San Carlos, CA, USA).

### Structural alignments and clustering

Global similarities between the present crystal structures (minus His tag residues) and those in the Protein Data Bank were identified using the EBI secondary-structure matching tool.^80^ Subsequent structural alignments were performed with CE (pairwise)^81^ and CE-MC (multiple).^82^ A pairwise distance matrix was derived using a loop-based Hausdorff measure (LHM) of structural dissimilarity,^31^ and proteins were clustered using the unweighted neighbour-joining method, UNJ,^83^ as implemented in T-REX.^84^

### Protein Data Bank accession codes

The coordinates and structure factors of RNase ZF-1a and RNase ZF-3e have been deposited in the RCSB Protein Data Bank under accession codes 2VQ8 and 2VQ9, respectively.

## Figures and Tables

**Table 1:** Zebrafish RNase variants

RNase	Breeding line	cDNA source^a^	No. copies	Representative example	
				Clone ID	GenBank ID
ZF-1a	Mixed	RZPD609	1	IMAGE:3716043^b^^,^^c^	AI476973
ZF-1b	SJD	SJD 5d embryo	1	IMAGE:5628280^c^	BM533038
ZF-1c	SJD	SJD 5d embryo	1	IMAGE:5627130^c^	BM573599
ZF-2a	TU	–d	1	–	AL928685:89541–89993
	TU	FDR107	> 100	FDR107-P00072-BR_P04	EH536003
	TU	NIH_ZGC_7	1	IMAGE:7270177	BC133890, CN510408
	AB	NIH_ZGC_8	19	IMAGE:7411519	BC110104, CO812928
	AB	*ad hoc*	1	–e	EF382670
	Mixed	RZPD609	1	IMAGE:3733625^b^	BF717998
ZF-2b	AB	NIH_ZGC_8	1	IMAGE:7410112^c^	BC092908, CO798809
	AB	NIH_ZGC_16	1	IMAGE:7213476	BC129341, CK866751
ZF-2c	AB	NIH_ZGC_8	1	IMAGE:7264666^c^	BC105745, CN326043
ZF-2d	AB	NIH_ZGC_8	1	IMAGE:7250488^c^	BC142860, CN170062
ZF-3a	TU	–d	1	–	BX465197:31652–32098
	TU	NIH_ZGC_10	1	IMAGE:8008790	DT070465
ZF-3b	Unspecified	*ad hoc*	1	–b	–
ZF-3c	AB	*ad hoc*	1	–e	EF382669
ZF-3d	TU	FDR107	17	FDR107-P00029-BR_C05	EH493831
	TU	NIH_ZGC_10	3	IMAGE:7047538^c^	CK142774
	TU	NIH_ZGC_7	1	IMAGE:7054926	CK026631
	AB	NIH_ZGC_16	13	IMAGE:7213270	CK869816
	AB	NIH_ZGC_8	2	IMAGE:7250821	CN170899
	AB	NIH_ZGC_20	1	IMAGE:7292890	CO250046
	Singapore	GISZF001	1	IMAGE:7151363^c^	CK688316
ZF-3e	Singapore	GISZF001	1	IMAGE:6907218^c^	CB363875
ZF-4	TU	–d	1	–	AL928685:53649–54053
	AB	*ad hoc*	1	–e	EF382671

a Libraries RZPD609 and NIH_ZGC_7 are normalized, all others are not. Specifics of the various libraries are as follows: FDR107, gut and internal organs (adult); GISZF001, whole body (embryo); NIH_ZGC_7, whole body (adult); NIH_ZGC_8, liver (adult); NIH_ZGC_10, whole body (adult); NIH_ZGC_16, gut (adult); NIH_ZGC_20, mixed tissue (adult); RZPD609, whole body (embryo, late somatogenesis) + liver (adult); SJD 5d embryo, whole body (embryo, 5 days). FDR107 and GISZF001 were constructed at the Genome Institute of Singapore (unpublished), NIH_ZGC libraries by the Mammalian Gene Collection Program team,^46,87^ RZPD609 by Clark *et al.*^19^ and SJD 5d embryo by the WashU Zebrafish EST Project team (unpublished). Further details are listed at http://www.ncbi.nlm.nih.gov/UniGene/lbrowse2.cgi?TAXID=7955&CUTOFF=D0.

b Data from Pizzo *et al.*^2^

c Resequenced during the course of this work.

d Data from the genomic assembly.

e Data from Cho and Zhang.^3^

**Table 2:** Potential hydrogen bonds in the B_1_ subsite and C-terminal regions

RNase A·Uvan^a^		RNase ZF-3e		hAng^b^		RNase ZF-1a	
Bond	Length (Å)	Bond	Length (Å)	Bond	Length (Å)	Bond	Length (Å)
A. *Pyrimidine binding and mimicry*							
Thr45 N–O2 Ura	2.88	Thr49 N–Cl CL1	3.18	Thr44 N–O^ε1^ Gln117	2.87	Thr51 N–O^ε2^ Glu122	2.91
Ura N3–O^γ1^ Thr45	2.79	Wat79 O–O^γ1^ Thr49	3.07	Gln117 N^ε2^–O^γ1^ Thr44	3.09	Thr51 O^γ1^–O^ε1^ Glu122	2.64
Thr45 O^γ1^–O^δ1^ Asp83	2.71	Thr49 O^γ1^–O^γ1^ Thr86	3.42	Thr44 O^γ1^–O^γ1^ Thr80	2.84	–	–
His12 N^δ1^–O Thr45	2.69	His16 N^δ1^–O Thr49	2.91	His13 N^δ1^–O Thr44	2.93	His16 N^δ1^–O Thr51	2.80

B. *Stabilisation of C-terminal conformation*							
(a) Interactions with external regions							
Ile107 N–O Ala122	2.73	Val110 N–O Arg123	2.80	–	–	–	–
Ala122 N–O Ile107	3.26	Arg123 N–O Val110	3.37	–	–	–	–
His105 N–O Val124	2.82	Lys108 N–O Val125	2.99	–	–	–	–
Val124 N–O His105	2.78	Val125 N–O Lys108	2.90	–	–	–	–
Lys66 N–O^δ2^ Asp121	2.81	–	–	–	–	–	–
Lys66 N^ζ^–O Asp121	2.67	–	–	–	–	–	–
–	–	Arg107 N^η1^–O Gly124	2.88	–	–	Arg106 N^η1^–O^δ1^ Asp123	2.92
–	–	–	–	–	–	Arg106 N^η2^–O^δ2^ Asp123	2.96
–	–	–	–	–	–	Lys85 N^ζ^–O^δ2^ Asp123	3.18
–	–	–	–	–	–	Lys85 N^ζ^–O^δ1^ Asn126	2.77
(b) Internal interactions							
His119 N^ε2^–O^δ1^ Asp121	2.66	–	–	–	–	–	–
–	–	–	–	Phe120 N–O Gln117	2.97	–	–
–	–	–	–	Arg121 N–O Ser118	3.14	Asn126 N^δ2^–O Asp123	2.73
–	–	–	–	Ser118 N–O^δ1^ Asp116	3.07	–	–
–	–	–	–	Ser118 O^γ^–O^δ1^ Asp116	2.46	–	–

Potential hydrogen bonds were identified with HBPLUS^88^ using default criteria (D–H···A angle > 90°, H···A distance < 2.5 Å).

a PDB entry 1RUV. ^86^

b PDB entry 1B1I.^21^

**Table 3:** Crystallographic statistics

	RNase ZF-1a	RNase ZF-3e
A. *Diffraction data*		
Space group	*P*2_1_	*I*222
Unit cell parameters		
*a* (Å)	33.2	43.2
*b* (Å)	39.4	61.0
*c* (Å)	46.1	115.2
β (deg)	98.8	
Resolution range (Å)	50–1.35	50–1.85
No. reflections measured	188,114	189,062
No. unique reflections	27,012	13,420
*R*_symm_^a^^b^	0.043 (0.313)	0.115 (0.248)
*I*/σ(*I*)	22.8 (2.0)	14.5 (5.1)
Completeness (%)	93.7 (61.8)	98.8 (90.6)

B. *Refined model*		
*R*_cry*st*_^b^	0.185	0.208
*R*_free_^c^	0.226	0.260
Deviation from ideality (r.m.s.)		
Bond lengths (Å)	0.007	0.013
Bond angles (deg)	1.37	1.83
No. atoms		
Protein	1065	982
Water	159	85
Chloride	1	2
Mean *B*-factor (Å^2^)		
Protein	10.0	28.2
Water	19.9	34.7
Chloride	45.8	27.4

Values in parentheses refer to the outermost shell (1.40–1.35Å and 1.92–1.85 Å for RNase ZF-1a and RNase ZF-3e, respectively).

a *R*_symm_ = ∑_*h*_∑_*i*_[|*I*_*i*_(*h*)–〈*I*(*h*)〉|/∑_*h*_∑_*i*_*I*_*i*_(*h*)], where *I*_*i*_ is the *i*th measurement and 〈*I*(*h*)〉 is the weighted mean of all measurements of *I*(*h*).

b *R*_cryst_ = ∑_*h*_|*F*_o_–*F*_c_|/∑_*h*_*F*_o_, where *F*_o_ and *F*_c_ are the observed and calculated structure factor amplitudes of reflection *h*, respectively.

c *R*_free_ is equal to *R*_cryst_ for a randomly selected 5 % subset of reflections not used in the refinement.^74^

## Footnotes

‡ http://zfin.org/

§ http://pymol.sourceforge.net/
